# Investigation of *PRKN* Mutations in Levodopa-Induced Dyskinesia in Parkinson’s Disease Treatment

**DOI:** 10.3390/biomedicines11082230

**Published:** 2023-08-09

**Authors:** Ana Gabrielle Bispo, Caio S. Silva, Camille Sena-dos-Santos, Dafne Dalledone Moura, Brenda Hanae Bentes Koshimoto, Bruno Lopes Santos-Lobato, Ândrea Ribeiro-dos-Santos, Giovanna C. Cavalcante

**Affiliations:** 1 Laboratory of Human and Medical Genetics, Federal University of Pará, Belém 66075-110, Brazil; bispogabrielle3@gmail.com (A.G.B.); scaio@hotmail.com (C.S.S.); camillebiologia@gmail.com (C.S.-d.-S.); akelyufpa@gmail.com (Â.R.-d.-S.); 2 Laboratory of Experimental Neuropathology, Federal University of Pará, Belém 66075-110, Brazil; daf.dalledone@gmail.com (D.D.M.); brendabentes01@gmail.com (B.H.B.K.); bruls4@ufpa.br (B.L.S.-L.)

**Keywords:** Parkinson’s disease, mitophagy, levodopa

## Abstract

Mitophagy is an important process that participates in mitochondrial quality control. Dysfunctions in this process can be caused by mutations in genes like *PRKN* and are associated with the development and progression of Parkinson’s Disease (PD). The most used drug in the treatment of PD is levodopa (LD), but it can cause adverse effects, such as dyskinesia. Currently, few studies are searching for biomarkers for an effective use of lLD for this disease, especially regarding mitophagy genetics. Thus, this work investigates the association of 14 variants of the *PRKN* gene with LD in the treatment of PD. We recruited 70 patients with PD undergoing treatment with LD (39 without dyskinesia and 31 with dyskinesia). Genotyping was based on Sanger sequencing. Our results reinforce that age at onset of symptoms, duration of PD, and treatment and dosage of LD can influence the occurrence of dyskinesia but not the investigated *PRKN* variants. The perspective presented here of variants of mitophagy-related genes in the context of treatment with LD is still underexplored, although an association has been indicated in previous studies. We suggest that other variants in *PRKN* or in other mitophagy genes may participate in the development of levodopa-induced dyskinesia in PD treatment.

## 1. Introduction

Mitochondria are organelles that perform important cellular functions, including ATP synthesis through oxidative phosphorylation (OXPHOS), control of reactive oxygen species (ROS), regulation of oxidative stress, and intracellular signaling [1,2,3]. Alterations in the quality control system of mitochondria are related to several neurodegenerative diseases, due to the accumulation of aged and dysfunctional mitochondria, which may increase ROS production and oxidative stress and generate synaptic and neuronal loss [2,4].

Among the cellular mechanisms that maintain mitochondrial homeostasis, mitophagy stands out, consisting of the process of degradation and removal of mitochondria considered dysfunctional, to prevent them from being in excess [5,6]. The impairment of the mitophagy process has already been widely described as a factor related to different diseases, including Parkinson’s Disease (PD) [3,7,8,9]. Because mitochondrial dysfunction resulting from alterations in genes related to mitophagy, such as *LRRK2*, *DJ-1*, *PINK1,* and *PRKN,* can lead to the production of ROS and aggregation of α-synuclein (major component of Lewy bodies, pathological hallmarks, and typically found in PD), these genes might be involved in promoting the neurodegeneration observed in PD [10]. Mitophagy is carried out mainly by the *PINK1* and *PRKN* genes, which, when mutated, can lead to irregularities in mitophagy and consequently the selective death of neurons, such as dopaminergic neurons in PD.

In this study, we highlight the *PRKN* gene (parkin RBR E3 ubiquitin protein ligase), also known as *PARK2* or Parkin, being the gene most associated with autosomal recessive PD of early onset [11,12,13]. It encodes Parkin, a protein that regulates mitophagy and mitochondrial biogenesis, participating in the quality control system of mitochondria [5,11]. A study carried out with *Drosophila* revealed that deficiencies in *PRKN* lead to the accumulation of dysfunctional mitochondria in several regions, including in dopaminergic neurons. Furthermore, an association between mitophagy failure and inflammation is suggested, through activation of the STING pathway (interferon gene stimulator), which may lead to the loss of dopaminergic neurons [14,15]. Therefore, defects in Parkin-mediated mitophagy are pertinent to PD [5,11,14].

Parkinson’s disease is characterized by the progressive loss of dopaminergic neurons in the substantia nigra of the central nervous system and by the accumulation of misfolded intracellular a-synuclein, leading to impaired muscle function, which causes symptoms such as bradykinesia, tremors, postural instability, and muscle rigidity, as well as non-motor symptoms such as depression, cognitive impairment, dementia, and anxiety [16,17,18,19]. Considered the second most common neurodegenerative disorder worldwide, it is estimated that 1% to 2% of the world’s population over 65 years of age is affected with PD [20].

Despite PD being widely studied, there are still no biomarkers that could indicate the early onset of PD and there are currently few forms of treatment for this disease, such as anticholinergics, amantadine (NMDA antagonist), monoamine oxidase inhibitors (MAOIs), catechol-O-methyl transferase inhibitors (COMTIs), and dopamine agonists [17]. The existing ones are aimed only at reducing the symptoms and not at containing the progression of the disease, in addition to having lower efficacy as neurodegeneration progresses, which represents an impact on the prognosis and quality of life of patients affected with PD [7,14].

In this context, levodopa (LD), a dopaminergic precursor, is the most used drug in the treatment of people affected by PD. LD is a prodrug, metabolized into dopamine when crossing the blood–brain barrier, thus supplementing the endogenous levels of this neurotransmitter, and, compared to other PD drugs, it is the most effective, as it has significant effects for improving symptoms [7,17]. Importantly, it relieves symptoms but does not prevent disease progression, and the prolonged use is related to adverse reactions, such as motor complications, among them motor fluctuations and dyskinesia. Levodopa-induced dyskinesia (LID) is characterized by movement disorders that include dystonia, myoclonus, chorea, and athetosis [21,22]. Undeniably, this condition also impairs the quality of life of patients with PD, who over time have worsening and debilitating symptoms.

Although there are many studies that suggest that mitochondrial dysfunction is essential in PD, there are many studies about LID in PD, and the relationship between mitophagy and PD is highly recognized, more recently in a therapeutic context [7,23,24,25], no studies are found in the global literature on mitophagy specifically in the treatment of LD. Thus, because genetic alterations can lead to dysfunctions in mitophagy, which in turn are related to PD, it is important to investigate potential biomarkers from the perspective of this mitochondrial process. Thus, the present work aims to investigate variants in *PRKN*, a gene related to mitophagy, in patients with PD who are treated with LD, seeking possible biomarkers that could be associated with therapeutic success and adverse reactions.

## 2. Materials and Methods

### 2.1. Sampling

For this research, 70 individuals with Parkinson’s Disease were selected, recruited from the Hospital Ophir Loyola, in the North region of Brazil. These patients were treated with levodopa in a multidrug therapy, of which 39 did not have LID, composing the control group, and 31 did have LID, composing the case group. All patients were at least 42 years of age at diagnosis and were divided into sex-matched groups. In addition, the use of levodopa in the treatment of these patients is equal to or greater than two years.

### 2.2. Selection of Variants

Through the public databases of the National Center for Biotechnology Information (NCBI) and a literature search, we selected the intronic variants rs9458609 and rs6935164 of the *PRKN* gene with relevant minor allele frequencies (MAF = 0.3 and MAF = 0.5, respectively) and with the potential to influence the development of diseases [26,27,28] but which have not yet been studied in relation to PD. From the gnomAD browser platform [29], closely located variants were selected for rs9458609 (rs1446954927, rs1562397973, rs1188406822, rs897123960, rs1469130556, rs1037288938 and rs1453267522) and for rs6935164 (rs1460805773, rs972410489, rs1372726016, rs187267734, rs182928580), all present in the *PRKN* gene.

### 2.3. DNA Extraction and Quantification

DNA was extracted from peripheral blood samples using the phenol-chloroform method, based on [30]. Quantification of the extracted DNA was performed using the NanoDrop 1000 spectrophotometer (Thermo Fisher Scientific Inc., Wilmington, DE, USA), to ensure quality of the extracted DNA.

### 2.4. Genotyping

Sequences of interest from the extracted DNA were amplified by PCR using specific primer pairs: for rs9458609 and close variants, 5′GAGTGATTCAGTCCAAGGCT3′ 5′TCAACAGTCAGTAAAAGGCAAC′, and for rs6935164 and close variants, 5′AAAGATTGCAGTGATGTGGC3′ and 5′AGAACTTCCACTTGAGCTGA3′. Table 1 presents the 14 analyzed variants, with their allelic changes and population frequency.

The PCR protocol was based on the work by Ferraz et al. (2022) [31]. For both primer pairs, the PCR was performed from a reaction with a volume of 20 µL, considering dNTP, 0.2 μL of Taq polymerase, 3.0 μL of DNA, and 11.3 μL of water. A Veriti thermal cycler (Thermo Fisher Scientific) was used for the reaction, through the program of 1 cycle of denaturation at 95 °C for 10 min, 35 cycles of denaturation at 95 °C for 15 s, the annealing temperature adjusted by the primer for 30 s, and extension at 72 °C for 1 min and 30 s.

Then, the PCR products were sequenced using the BigDye Terminator Sequencing Kit version 3.1 (Thermo Fisher Scientific) in an ABI PRISM 3130 Genetic Analyzer (Thermo Fisher Scientific). Nucleotide sequences generated by the DNA sequencer were traced by Sequencing Analysis Software version 5.2 (Thermo Fisher Scientific). The submitted genotyping was read using Clustal Omega [32,33] and the chromatogram analysis was performed with Chromas software version 2.6.6 (https://technelysium.com.au/wp/).

### 2.5. Genomic Ancestry

Brazil has a population with diverse contributions of genomic ancestry, especially Native American (NAM), European (EUR), and African (AFR), because of the complex process of the population formation. In this context, to avoid possible bias in the interpretation of results due to population substructuring, we employed a set of ancestry informative markers (AIM) previously developed and established by our research group [34,35,36].

### 2.6. Statistical Analysis

Statistical analyses and graphs were performed using the JASP [37] and R [38], with *p* values < 0.05 being considered statistically significant.

## 3. Results

### 3.1. Characterization of the Cohort

The studied cohort consisted of 70 individuals with PD undergoing treatment with levodopa, in which the case group represents individuals with dyskinesia and the control group represents individuals without dyskinesia. We found that age at onset of symptoms, duration of PD and treatment, and LD dosage were factors that showed statistically significant differences between the two groups (Table 2). On the other hand, we found no significant differences between sex, genomic ancestry, family history of PD, the primary symptom, and UPDRS.

**Figure 1:**
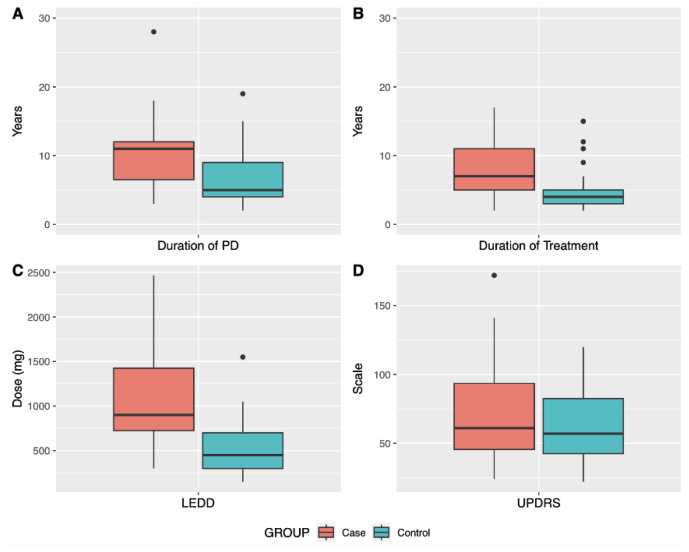
Clinical variables related to the treatment of patients with levodopa with and without dyskinesia (case and control, respectively). (**A**): Duration in years of Parkinson’s Disease for patients in both groups; (**B**): Duration in years of treatment with levodopa; (**C**): Dosage in mg of levodopa; (**D**): UPDRS PD rating scale.

Figure 1 shows that, for patients with dyskinesia, duration of both PD and treatment are longer compared to patients without dyskinesia. Likewise, the dosage of levodopa (LEDD) increases with therapeutic time. These results were expected considering that prolonged treatment with LD can lead to the development of dyskinesia, but these variables were controlled in the statistical analysis. Regarding the UPDRS international scale, there was no statistically significant difference, indicating that both groups would be in similar motor stages of the disease. This scale was proposed in the 1980s and its revised version is the most widely used tool in the world to aid in the diagnosis of PD [39,40].

**Figure 2:**
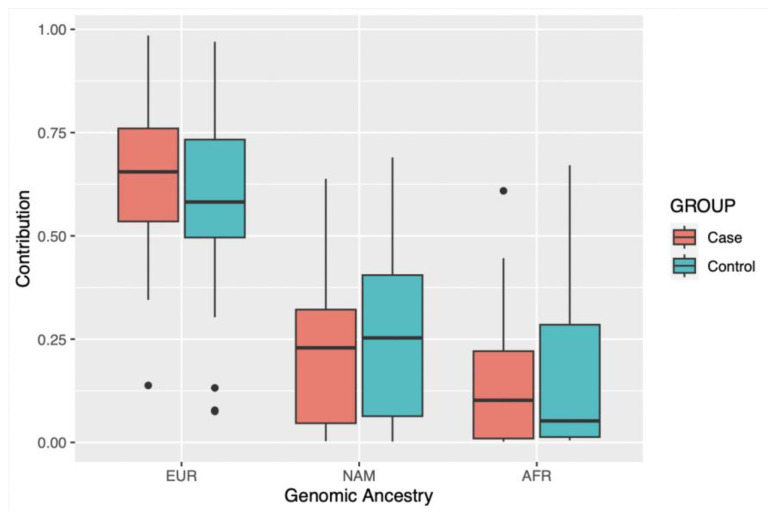
Contribution of each genomic ancestry investigated in the studied groups. EUR: European; NAM: Native American; AFR: African.

When analyzing the average contribution of genomic ancestry of the study cohort, which is composed of individuals from a population of the Brazilian Amazon, it is observed that the greatest contribution is European, followed by Native American and African, for both groups (Figure 2). This result corroborates previous studies carried out in the same region [31,41].

### 3.2. Analysis of Variants

Then, the distribution of variants and their allelic and genotypic frequencies between groups were analyzed. All groups are in Hardy–Weinberg Equilibrium (*p* > 0.05). Only the rs9458609 and rs6935164 variants showed genotype variation in the studied cohort; the other 12 variants presented only the homozygous genotype for the reference allele, reinforcing the previously described MAF. The frequencies of these two variants for our cohort are shown in Table 3. Furthermore, when we analyzed the dependence of the variants in relation to the sex of the patients, we did not observe statistically significant differences.

## 4. Discussion

Parkinson’s disease is characterized by the progressive loss of dopaminergic neurons, which leads to motor and non-motor symptoms. Mutations in genes such as *PINK1*, *PRKN*, *DJ-1*, *ATP13A2*, *LRRK2*, *SNCA,* and *VPS35* are commonly found in PD, and dysfunctions in these genes affect essential processes in the mitochondrial quality control system that ensure mitochondrial function, like fusion and fission, biogenesis, and mitophagy [5,7]. The role of mitochondria in the neurodegenerative process of PD has been increasingly described. Mitochondrial dysfunction results in multiple effects, such as decreased ATP generation, increased ROS, and dysregulation of Ca^2+^ levels. These disorders lead to impaired proteostasis in neurons, hence the accumulation of proteins such as a-synuclein and a reduction in axonal transport to vesicles and mitochondria, resulting in neurodegeneration and neuroinflammation [42].

In this sense, mitophagy is a crucial mechanism in mitochondrial quality control and is a key process in neurodegeneration [43] so that mutations in genes responsible for mitophagy, such as *PRKN*, can lead to the onset and progression of diseases, including PD. The drug most used for the treatment of PD is levodopa, but its prolonged administration can cause adverse effects, such as dyskinesia, characterized by involuntary muscle movements, which affects the quality of life of these individuals [7,21]. One study showed that after 15 years of treatment with levodopa, 94% of patients developed dyskinesia [44], which demonstrates the need for studies that investigate this adverse effect and suggest effective biomarkers to improve quality of life of PD patients.

Among the different genes involved in the complex network of PD, the *PRKN* gene is most associated with the development of this disease [3,7,10]. However, a study [45] demonstrated that individuals with certain *PRKN* mutations have a better response to LD treatment, although they are possibly at a higher risk of developing dyskinesia compared to individuals without these *PRKN* mutations. In fact, recently, it has been suggested that mutations in *PRKN* could influence the onset of LID at an early stage, although this relationship has not yet been much explored [13,46].

Thus, in the present study, the distribution of 14 variants in the *PRKN* gene of the mitophagy pathway was investigated in a cohort of PD patients undergoing treatment with LD, divided into individuals with and without dyskinesia. The study looked for two mutations in this gene that are currently understudied but which have relevant MAF (rs9458609 and rs6935164) in addition to 12 closely located variants in *PRKN*.

No statistically significant association was found for rs9458609 and rs6935164 between the groups with and without dyskinesia, suggesting that these mutations may not be involved with the development of this adverse effect in patients with PD, and these variants do not seem to be dependent on the sex of the patients.

The rs9458609 variant was previously mentioned in [26], who, through a *PRKN* and *PACRG* mapping, significantly related this variant to leprosy in Indian patients. More recently, this variant has been reported in linkage disequilibrium with a polymorphism of the same gene, rs1801582 (r2 = 0.06) [28], which, in turn, had already been associated with susceptibility to the development of PD [47], although this result was not observed in a study on PD in another population [48]. As far as we know, the other variants analyzed here had not been reported in the literature until the present study.

In addition, demographic and clinical characteristics related to the treatment of these patients were also investigated. As expected, statistically significant differences were identified between groups comparing age of symptom onset, duration of PD, duration of treatment, and levodopa equivalent daily dose (LEDD). One study highlighted that the age of onset of PD is a determinant aspect of the risk of dyskinesia, whereas patients with 5 years of treatment and age of onset between 40 and 49 years had a 46% higher risk of developing dyskinesia than individuals aged 70 to 79 years at onset of PD [49]. Also, it has already been described that disease progression, younger age at onset of PD, and dose and treatment period are predictive factors for the development of dyskinesia [22,50]. It was demonstrated in a previous study [51] that the female individuals and also those with the most advanced form of the disease, that is, higher scores on the UPDRS, have a greater risk of dyskinesia; however, our analysis did not indicate a statistically significant difference between the groups when observing the sexes and the UPDRS. Regarding genomic ancestry as a risk factor for LID, the same study pointed to individuals from the geographic region of North America as a risk factor compared to Europe. In our study, there were no statistically significant differences when observing the contributions of genomic ancestry (European, Native American, and African) in the investigated population. Currently, there are few studies that assess genomic ancestry as a risk factor for LID, especially in Brazil, which reinforces the relevance of the present work.

## 5. Conclusions

In short, our results highlight that age at onset of symptoms, duration of PD and treatment, and LD dosage are factors that may influence the occurrence of dyskinesia, but the variants investigated here may not be involved with this adverse effect, suggesting that studies in larger cohorts with the same variants, particularly rs6935164, should be conducted to reinforce our findings, but also that other genetic factors of *PRKN* and mitophagy in general may play a role in LID in patients with PD.

## Figures and Tables

**Table 1:** Description of the investigated *PRKN* gene variants.

Variant	Alleles	MAF
rs9458609	A>G	0.30678
rs1446954927	A>G	0.00007
rs1562397973	G>A	0.000007
rs1188406822	G>A	0.000007
rs897123960	T>C	0.000014
rs1469130556	A>G	0.00007
rs1037288938	G>A	0.000021
rs1453267522	T>G	0.000014
rs6935164	A>G	0.497471
rs1460805773	T>G	0.000007
rs972410489	G>C	0.000007
rs1372726016	G>A	0.000007
rs187267734	C>A	0.000007
rs182928580	G>A	0.003313

**Table 2:** Data from the case group (PD patients who developed dyskinesia) and the control group (PD patients without dyskinesia), both undergoing treatment with LD.

Variable	Case	Control	*p*-Value
*n*	31	39	
Age of onset of symptoms, years ^a^	47.5 ± 1.68	55.3 ± 1.55	0.001
Sex, % male/female ^b^	74.2/25.8	61.5/38.5	0.263
European ancestry ^c^	0.643 ± 0.036	0.588 ± 0.035	0.309
Native American ancestry ^c^	0.214 ± 0.031	0.259 ± 0.033	0.350
African ancestry ^c^	0.143 ± 0.029	0.154 ± 0.031	0.692
Family history, % yes/no ^b^	17.2/82.8	30.6/69.4	0.215
Primary Symptom, % tremor/others ^b^	48.4/51.6	66.7/33.3	0.123
Duration of PD ^d^	10.0 ± 0.92	6.9 ± 0.62	0.0037
Duration of treatment ^d^	8.1 ± 0.68	4.97 ± 0.51	8.1 × 10^−5^
LEDD ^d^	1066.1 ± 94.7	540.2 ± 44.7	1.8 × 10^−6^
UPDRS ^d^	73.2 ± 6.5	64.3 ± 4.3	0.370

^a^ Mean value ± SE (Standard Error of Mean), Student’s *t*-test; ^b^ Values in distribution percentages, chi-squared test ^c^ Mean ± SE values, Mann–Whitney test. ^d^ Mean ± SE values, Wilcoxon test. LEDD: Levodopa Equivalent Daily Dose. UPDRS: Unified Parkinson’s Disease Rating Scale.

**Table 3:** Distribution of allele and genotypic frequencies of variants rs9458609 and rs6935164 for PD patients with dyskinesia (case) and without dyskinesia (control).

Variant	Genotype	Case (%)	Control (%)	*p*-Value ^a^	OR (95%CI) ^b^
rs9458609		*n* = 29	*n* = 36		
	AA	16 (55.2)	17 (47.2)	0.773	0.817 (0.207–3.229)
	AG	9 (31.0)	17 (47.2)	0.807	0.847 (0.224–3.203)
	GG	4 (13.8)	2 (5.6)	0.330	3.306 (0.299–36.601)
		A = 0.7/G = 0.3	A = 0.7/G = 0.3		
rs6935164		*n* = 28	*n* = 30		
	AA	6 (21.4)	13 (43.3)	0.062	0.229 (0.049–1.079)
	AG	12 (42.8)	10 (33.3)	0.293	2.320 (0.483–11.148)
	GG	10 (35.8)	7 (23.4)	0.287	2.333 (0.491–11.080)
		A = 0.4/G = 0.6	A = 0.6/G = 0.4		

^a^ *p*-value obtained by logistic regression with correction for confounding factors: age at onset of symptoms, duration of PD, duration of treatment and LEDD. ^b^ Odds Ratio (OR) and 95% Confidence Interval (95%CI) obtained by logistic regression.

## Data Availability

The dataset generated and analyzed for this study can be found in the FigShare repository (https://doi.org/10.6084/m9.figshare.21864717), accessed on 26 June 2023.
